# Allele-Level Haplotype Frequencies and Pairwise Linkage Disequilibrium for 14 KIR Loci in 506 European-American Individuals

**DOI:** 10.1371/journal.pone.0047491

**Published:** 2012-11-05

**Authors:** Cynthia Vierra-Green, David Roe, Lihua Hou, Carolyn Katovich Hurley, Raja Rajalingam, Elaine Reed, Tatiana Lebedeva, Neng Yu, Mary Stewart, Harriet Noreen, Jill A. Hollenbach, Lisbeth A. Guethlein, Tao Wang, Stephen Spellman, Martin Maiers

**Affiliations:** 1 Center for International Blood and Marrow Transplant Research, Minneapolis, Minnesota, United States of America; 2 National Marrow Donor Program, Minneapolis, Minnesota, United States of America; 3 Departments of Oncology and Pediatrics, Georgetown University, Washington D.C., United States of America; 4 UCLA Immunogenetics Center, Department of Pathology and Laboratory Medicine, David Geffen School of Medicine at UCLA, University of California Los Angeles, Los Angeles, California, United States of America; 5 American Red Cross, HLA Laboratory, Dedham, Massachussets, United States of America; 6 University of Minnesota Medical Center, Fairview, Minneapolis, Minnesota, United States of America; 7 Children’s Hospital Oakland Research Institute, Oakland, California, United States of America; 8 Stanford University, Stanford, California, United States of America; 9 Center for International Blood and Marrow Transplant Research, Milwaukee, Wisconsin, United States of America; University of Alabama at Birmingham, United States of America

## Abstract

The immune responses of natural killer cells are regulated, in part, by killer cell immunoglobulin-like receptors (KIR). The 16 closely-related genes in the KIR gene system have been diversified by gene duplication and unequal crossing over, thereby generating haplotypes with variation in gene copy number. Allelic variation also contributes to diversity within the complex. In this study, we estimated allele-level haplotype frequencies and pairwise linkage disequilibrium statistics for 14 KIR loci. The typing utilized multiple methodologies by four laboratories to provide at least 2x coverage for each allele. The computational methods generated maximum-likelihood estimates of allele-level haplotypes. Our results indicate the most extensive allele diversity was observed for the KIR framework genes and for the genes localized to the telomeric region of the KIR A haplotype. Particular alleles of the stimulatory loci appear to be nearly fixed on specific, common haplotypes while many of the less frequent alleles of the inhibitory loci appeared on multiple haplotypes, some with common haplotype structures. Haplotype structures cA01 and/or tA01 predominate in this cohort, as has been observed in most populations worldwide. Linkage disequilibrium is high within the centromeric and telomeric haplotype regions but not between them and is particularly strong between centromeric gene pairs *KIR2DL5*∼*KIR2DS3S5* and *KIR2DS3S5*∼*KIR2DL1,* and telomeric *KIR3DL1*∼*KIR2DS4*. Although 93% of the individuals have unique pairs of full-length allelic haplotypes, large genomic blocks sharing specific sets of alleles are seen in the most frequent haplotypes. These high-resolution, high-quality haplotypes extend our basic knowledge of the KIR gene system and may be used to support clinical studies beyond single gene analysis.

## Introduction

The natural killer (NK) cell immunoglobulin-like receptor (*KIR*) genes are clustered in a ∼160 kilobase (kb) region in the leukocyte receptor gene complex on the long arm of human chromosome 19 [1], [2], [3]. The KIR gene complex consists of a centromeric region bordered by the genes *KIR3DL3* and *KIR3DP1* and a telomeric region bordered by *KIR2DL4* and *KIR3DL2*. Within each region, the KIR genes lie less than 3 kb apart. The head to tail orientation of the KIR genes and the similarity of their gene structures and sequences suggest that the 16 KIR genes arose by duplication events [1]. Six genes (*KIR2DS1, KIR2DS2, KIR2DS3, KIR2DS4, KIR2DS5, KIR3DS1*) encode proteins on the surface of NK cells with short (S) intracytoplasmic tails that are thought to be activating receptors [4]. Seven genes (*KIR2DL1, KIR2DL2, KIR2DL3, KIR2DL5, KIR3DL1, KIR3DL2,* and *KIR3DL3*) encode cell surface receptors with longer (L) cytoplasmic tails whose signals inhibit NK cell activation. *KIR2DL4* encodes a receptor that performs both inhibitory and activating functions. Two KIR are pseudogenes (*KIR2DP1, KIR3DP1*).

Studies of individuals, families, and populations have demonstrated that individuals vary in the number and type of KIR loci present in their genomes [5], [6], [7]. *KIR2DL1, KIR2DL2, KIR2DL3, KIR2DL5, KIR3DL1, KIR2DS1- KIR2DS5, KIR3DS1* are found in some (but not all) individuals. In contrast, the four loci marking the boundaries of the centromeric and telomeric KIR regions, *KIR3DL3, KIR3DP1, KIR2DL4*, and *KIR3DL2*, are found in essentially all individuals and are termed ‘framework’ genes. The variation in gene number and the gene positioning has been confirmed by long range genomic sequencing of 24 KIR haplotypes [1], [2], [8].

KIR gene-content haplotypes have historically been divided into two broad groups. Group A haplotypes, initially identified by the absence of a specific DNA restriction enzyme fragment, are currently defined by the presence of *KIR2DS4* as the only stimulatory KIR gene in combination with *KIR2DL1, KIR2DL3, KIR2DL4, KIR3DL1, KIR3DL2, KIR3DL3, KIR2DP1* and *KIR3DP1* [9]. Group B haplotypes carry other KIR genes (inhibitory: *KIR2DL2*, *KIR2DL5*; stimulatory: *KIR2DS1*, *KIR2DS2*, *KIR2DS3*, *KIR2DS4*, *KIR2DS5*, *KIR3DS1*), sometimes including Group A haplotype-specific KIRs. Diversity within the Group A and Group B haplotypes has been noted with respect to gene content [6], [10] as well as allelic variation [8], [9], [11], [12]. This variation is found within both centromeric and telomeric regions. Since strong linkage disequilibrium is observed within the centromeric and telomeric regions, but not between the two [9], [13], it has been suggested that a KIR haplotype consists of various permutations of centromeric and telomeric region variants [14].

Although not evident during the initial naming of KIR genes, it became appreciated later that some genes are actually alleles at the same locus, determined by their similar positioning in the gene complex [1] and their negative linkage disequilibrium [6]. Thus *KIR2DL2* and *KIR2DL3* are variants of the same locus, as are *KIR3DL1* and *KIR3DS1,* and *KIR2DS3* and *KIR2DS5*; these multiple names are sometimes combined (e.g., *KIR2DS3S5*). *KIR2DL5* and *KIR2DS3S5* have been duplicated and appear in two different positions in KIR haplotypes [15], [16]. *KIR2DL5* is always associated with either *KIR2DS3* or *KIR2DS5*. Specific combinations of alleles at each locus are commonly observed: KIR2DL5B*002 is adjacent to KIR2DS3*00103 in the centromeric region, while KIR2DL5A*001 (or *005) is adjacent to KIR2DS3*002 in the telomeric region [17]. Haplotypes carrying both copies of *KIR2DL5* and of *KIR2DS3 (*or *KIR2DS5*) have been observed in studies of European families, although haplotypes with only one of each paralog have also been observed [18], [19].

Other, less common haplotypes, apparently deriving from unequal recombination, have also been noted. A haplotype present in ∼4.5% of a sample of European individuals carries a duplicated segment bearing *KIR2DL4* and *KIR3DL1* [20], [21]. A chimeric gene formed at the site of recombination converts pseudogene, *KIR3DP1*, to an expressed locus [22]. Family and population studies and fosmid cloning have detected numerous other haplotypes with large deletions, duplications, and novel hybrid genes, such as a fusion of the *KIR3DL1* and *KIR3DL2* loci [19], [23], [24].

Previous work has demonstrated the important role of KIR in human health and disease. KIR participate in the interaction between maternal NK cells and fetal trophoblasts during fetal implantation [25]. Studies of disease susceptibility and resistance have noted associations with specific KIR loci [26]. In hematopoietic stem cell transplantation, specific KIR loci have been shown to have an impact on outcome, and adoptive therapy based on KIR/ligand reactivity has been used for the treatment of malignant relapse [27], [28], [29]. Given that KIR allelic products have been shown to differ in their specificity for and affinity of ligand binding [30], [31], these allelic differences may translate into differences in disease progression [26], [32], [33].

In order to better understand the genetic diversity of KIR and to evaluate its role in transplantation, we have estimated allele-level haplotype frequencies and pairwise linkage disequilibrium for the 14 functional KIR loci in 506 individuals with European ancestry. These estimates provide the first view in a large cohort of haplotypic associations of alleles of the KIR loci.

## Materials and Methods

### Subjects

DNA samples for a total of 506 unrelated individuals were obtained from the National Marrow Donor Program (NMDP) Research Repository. All individuals are of European ancestry and participated in a 10/10 HLA-matched hematopoietic stem cell transplantation (468 donors and 38 recipients) for acute lymphoblastic leukemia, acute myeloid leukemia, myelodysplastic syndrome, or chronic myelogenous leukemia. For each, KIR characteristics were unknown at the time of transplant. All subjects provided informed consent for participation in research. The NMDP Institutional Review Board approved the study.

### KIR Allele Identification

Allele-level genotyping focused on 14 KIR loci (*KIR2DL1–5*, *KIR2DS1–5*, *KIR3DL1–3* and *KIR3DS1*) and was performed in three independent laboratories. Participating laboratories utilized different genotyping methodologies, including sequence-specific-oligonucleotide (SSO) hybridization, sequence-specific-primer typing (SSP), and DNA sequencing [16], [17], [34], [35], [36], [37], [38], [39], [40], [41], [42], [43]. Results were interpreted to a three-digit allele designation using the Immuno Polymorphism Database- KIR version 1.2.0 or higher [44]. The majority (85%) of the samples were typed in duplicate by two different laboratories and final calls were obtained by consensus. Results from a fourth laboratory were used to resolve ambiguities and discrepancies. Each laboratory reported results using the Standard Reporting Format for KIR Genotyping Data (IPD) (http://www.ebi.ac.uk/ipd/kir/standards.html). This is an open format that distinguishes between haploid and diploid ambiguities, accepts all levels of resolution, and reports the observed number of loci. This format is also easily translated into XML for downstream storage and analysis.

Physical linkage of loci and an assay for copy number variation were employed to define haplotypes for 7.5% of individuals whose haplotypes were unresolved in the first stage of analysis (see below, haplotype estimation). Methods have been previously described [12]. Briefly, haplotype specific extraction (HSE) (Qiagen, Valencia, CA) was used to isolate a DNA fragment carrying a single allele at a locus. Once isolated, the alleles were identified by DNA sequencing for expressed genes and sequence-specific amplification for the pseudogenes and some fusion genes. An extended PCR and sequencing was used to link some loci. Alleles for the second KIR haplotype in an individual were defined as those alleles not assigned by physical linkage to the first haplotype. Amplification reactions were used to identify the presence of *KIR2DP1*, *KIR3DP1*, and fusion genes [19], [22], [23], [45], [46]. TaqMan copy number assays were used to measure *KIR2DL4* copy number variation (Applied Biosystems, Inc., Foster City, CA) [20].

### Identification of Novel Alleles

Novel alleles (Table S1) were isolated for DNA sequencing using allele specific amplification, by cloning, or by using HaploPrep reagents (Qiagen, Valencia, CA) as previously described [39], [40], [42], [43], [47], [48]. Two or three overlapping PCR amplicons were generated from each KIR gene covering the complete coding sequence. Sequences were compared to known KIR sequences obtained from the IPD-KIR database version 2.1.0 to determine allelic assignments. Allele designations for novel KIR alleles were assigned by the International Union of Immunological Societies and World Health Organization (IUIS/WHO) Subcommittee for KIR Nomenclature. Alleles have been deposited in GenBank and the Immuno Polymorphism Database.

### Determination of Reference Haplotypes

An implementation of the expectation-maximization (E-M) algorithm in the Haplo-IHP software [49] was used to confirm that each individual’s gene-content haplotypes could be explained by a pair from our set of reference haplotypes. The software defines gene content as zero or one copies for each gene.

**Figure 1:**
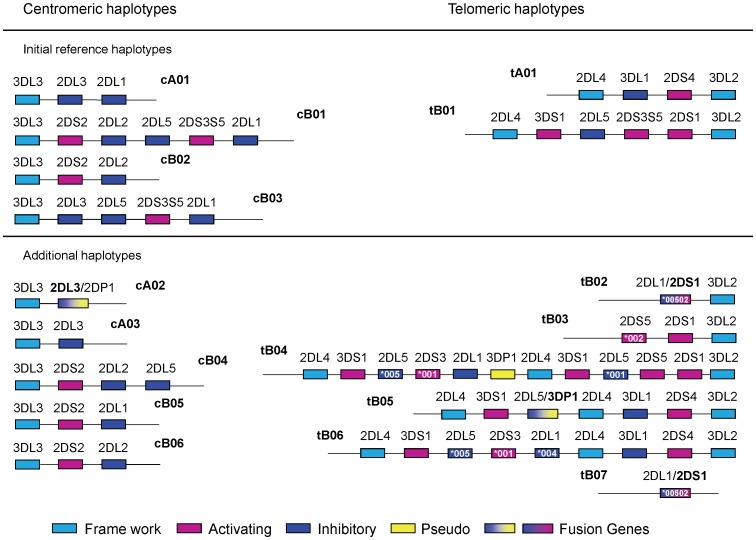
Reference structures for KIR haplotype estimation. The reference haplotypes used in the prediction model are shown as centromeric and telomeric haplotype structures (e.g., cA01 indicates centromeric A haplotype 01). The colors represent KIR gene characteristics: framework (light blue), activating (pink), inhibitory (dark blue and green), pseudogene (yellow), and fusion gene (gradient). For fusion genes, the assigned locus name is in bold. Genes represented by a single allele are labeled with the allele name. Pseudogenes are omitted except when they occur in a duplicated region or as a fusion gene. All but one haplotype (cA03∼tB07) have been previously reported [8], [13], [15], [19], [21], [22], [23], [24], [50].

The Haplo-IHP algorithm also requires as input a list of reference haplotypes. The seven gene-content haplotypes described in Pyo et al. [8] were used as the reference set for the initial analysis (Figure 1). The genotypes of 38 individuals (7.5%) were unresolved given this set of reference haplotypes. Additional laboratory analysis was performed for these individuals to determine gene-content haplotypes. This analysis included physical linkage of neighboring alleles and clarification of copy number for the *KIR2DL4* locus (selected to detect a common haplotype with a gene duplication [20], [21], [22]). Based on this analysis, five centromeric and six telomeric regions were added to the set of reference haplotypes (Figure 1).

Structural haplotype estimation was repeated with 15 reference haplotype structures, including 14 haplotypes consisting of previously reported centromeric and telomeric regions [8], [13], [15], [19], [21], [22], [23], [24], [50] and 1 novel haplotype (cA03∼tB07) identified through molecular characterization of the haplotype as described above. *KIR2DS3* and *KIR2DS5* were treated as a single gene (*KIR2DS3S5*) based on previous reports of their alternative and invariant linkage with *KIR2DL5* [15], [16].

The algorithm accepts constraint patterns for LD between loci and rejects individuals that do not meet the constraint patterns. Based on previous observations [6], [10], [13], [15] that are consistent with our set of reference haplotypes, we applied five locus-level haplotype constraints to confirm all individuals could be explained by our reference haplotypes: (1) framework gene *KIR3DL3* must be present, (2) *KIR2DL5* and *KIR2DS3S5* must be in complete linkage disequilibrium (LD) so that either both loci are present or both are absent, (3) *KIR3DL1* and *KIR2DS4* are in complete LD, and (4) *KIR2DL2* and *KIR2DL3* cannot both be present. It was not assumed that *KIR3DS1* and *KIR3DL1* are in complete negative LD based on [20], [21], [22]. All genotypes of all individuals were consistent with these constraints.

### Allelic Haplotype Estimation

We estimated allelic haplotype frequencies using an E-M algorithm and assigned the most likely haplotype pair for each individual. After determining that each individual’s genotype could be explained by at least two reference haplotypes, a custom E-M algorithm was used to estimate allelic haplotype frequencies, from which LD statistics were calculated. This implementation of the E-M algorithm does not support missing or unreported genotypes, but does support ambiguous allelic genotyping results and an arbitrary number of gene copies per haplotype (including explicit absence).

**Figure 2:**
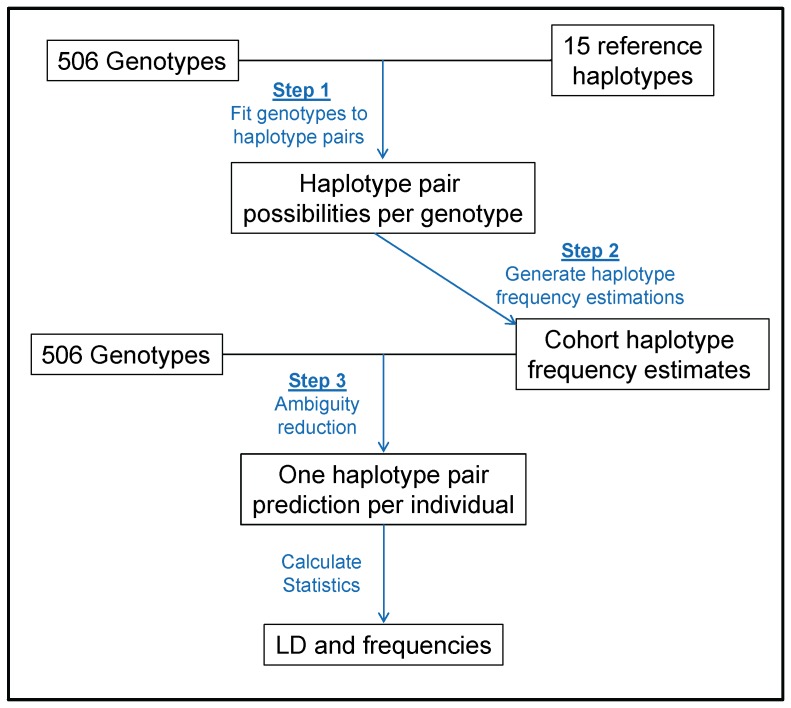
Overview of methods for estimation of haplotypes consisting of fully resolved alleles at each locus. Estimation was performed in three main steps. The first step uses the reference haplotype structures to generate all possible allelic haplotypes for each individual. The second step aggregates these individual haplotype possibilities across all individuals and uses an E-M algorithm to produce haplotype frequency estimates for the entire cohort. Step three utilizes these cohort-wide estimates to perform ambiguity reduction for each sample, producing a haplotype pair prediction for each individual. Further statistics are derived from these individual haplotype pair predictions.

An overview of the process is depicted in Figure 2 and details follow. The first step fits individual allelic genotypes to all possible pairs of allelic haplotypes. Given the 506 allelic genotypes and the 15 reference haplotypes, each genotype was expanded into all possible haplotype pairs. The output explicitly details the allelic and haplotypic ambiguities for each individual as a collection of allelic haplotype pairs. A decision matrix was created to address gene duplications on a haplotype (Table S2). The matrix mapped *KIR2DL1*, *KIR2DL5*, and *KIR2DS3S5* genotypes to their centromeric and telomeric locations based on the structure of known allelic haplotypes. A nomenclature system was used whereby paralogs were distinguished by appending a ‘C’ or ‘T’ to designate location in the centromeric or telomeric region, respectively; subsequently a number was added, (e.g., *KIR2DL5T1* and *KIR2DL5T2*) to accommodate additional gene duplications. The numbers provide a unique identity, but do not imply any relative location or order. This nomenclature, used in the E-M, appears in the decision matrix (Table S2) and the haplotype frequencies table (Table S3). Haplotypes were named according to previously recommended nomenclature standards http://www.ebi.ac.uk/ipd/kir/standards.html [8].

The next step aggregated the individual information to produce haplotype frequency estimations for the entire cohort. Within each individual, all haplotype pairs were given equal weight; for example, if four haplotype pairs could exhaustively explain an individual’s allelic genotype, each of the haplotype pairs would be given a fractional count of 0.25. These counts were aggregated per haplotype across all individuals and used as input to a custom E-M algorithm, which then estimated gene-content and allelic haplotype frequencies for the entire sample [51], [52].

The final step made an allelic haplotype pair assignment for each individual, given the individual allelic genotypes and the estimated sample-level haplotype frequencies.

When multiple haplotype pairs could explain an individual’s genotype, the pair with the highest total frequency was assigned. For homozygotes, the total frequency was calculated as the product of the two frequencies; for heterozygotes, the total frequency was twice the product of the two frequencies. This procedure resolved allele, copy number, and phase ambiguities on a per-sample basis.

### Calculation of Linkage Disequilibrium (LD) Statistics

Custom software and the ‘LDkl’ function of the Genetic Analysis Package (GAP) for the R language for statistical computing (R Core Development Team, 2009) were used to compute LD statistics between haplotype region (e.g., cA01, tB01, etc), locus and allele pairs. Overall LD between loci was assessed using the Wn statistic (identical to r^2^ for biallelic loci or Cramer’s V extension for multiple alleles) [53]; the D’ statistic was used to measure LD between regions and alleles and significance testing for allele pairs was accomplished via a standard chi-squared measure [54], [55], [56], [57].

### Analysis of Allele and Genotype Frequency Distributions

Watterson’s homozygosity statistic (F, used to examine allele frequency distributions relative to expectations under neutrality) and the fit of genotype frequencies to expectations under Hardy-Weinberg Equilibrium proportions were performed with the PyPop software framework for population genetics analysis [58], [59].

## Results

We estimated pairs of allelic full-length haplotypes for each individual. This allowed fine-grained analysis of genotypes, allele frequencies, haplotype frequencies, conserved haplotype blocks, and LD at the region, locus, and allele levels.

### Genotype Assignment

Genotyping for the 14 functional KIR loci in the study cohort yielded three-digit allele assignments for each locus present in each of the 506 individuals. Heterozygous results (two alleles identified at a locus in an individual) allowed confirmation of the presence of that locus on both chromosomes in that individual. However, for loci where only one allele was identified, the genotyping methods were unable to distinguish between homozygosity at the locus (present on both chromosomes), or, alternatively, absence of the locus on the second chromosome in that individual. In order to estimate copy number for each locus, posterior probabilities derived from the estimated haplotype frequencies were used to assign the most likely gene-content haplotypes (and therefore, full genotypes) for each individual.

### KIR Allele Frequency Distributions

**Figure 3:**
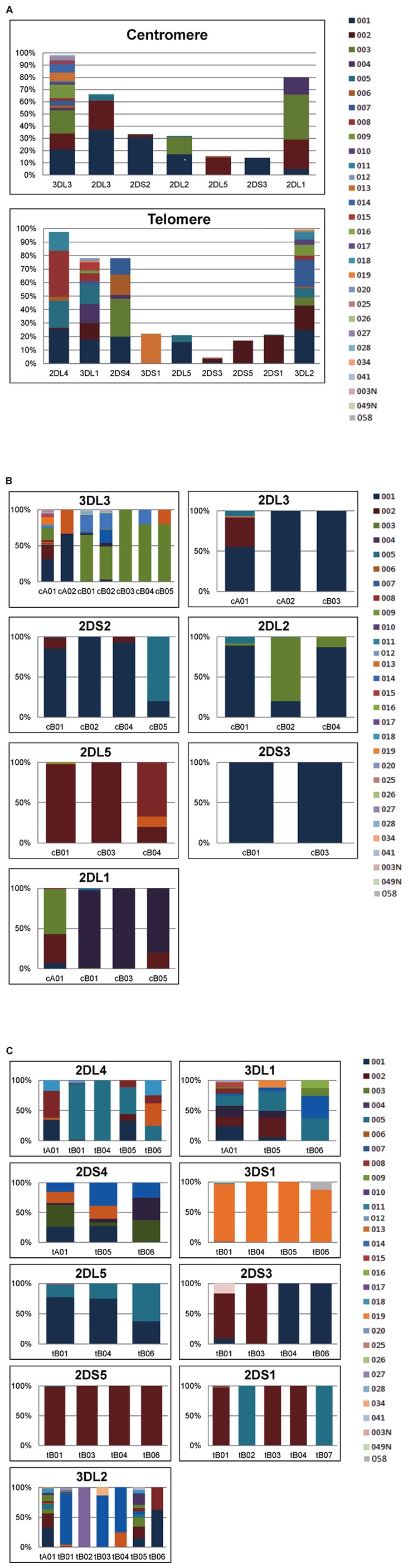
KIR gene and allele frequencies. The frequencies of genes in the centromeric and telomeric regions are provided for 1012 KIR haplotypes (3a). Multicolored segments represent the frequencies of alleles at each locus. Figures 3b (centromere) and 3c (telomere) depict the allele frequencies when present in the given gene-content haplotype. Duplicated loci have been excluded to conserve space.

Gene and allele frequencies for each KIR locus tested are shown in Figure 3 (details are in Table S4). The framework genes as well as the telomeric A genes (*KIR3DL1* and *KIR2DS4*) are most likely to be present on gene-content haplotypes and exhibit the most diversity. For example, *KIR3DL3* is found in 100% of KIR centromeric haplotypes; 24 alleles were found at frequencies varying from <1–21% at this locus. In general, the inhibitory loci (and framework genes in particular) exhibit more allelic variability than the stimulatory loci; however, the stimulatory loci are more variable in gene content. While not statistically significant, there is a strong trend toward more even distributions for *KIR2DL1*, *KIR2DL2L3* (*KIR2DL2* and *KIR2DL3*), *KIR2DL4* and *KIR3DL1S1* (*KIR3DL1* and *KIR3DS1*), all with normalized deviates of the F statistic ∼(−1). For the stimulatory loci, particular alleles appear to be nearly fixed on specific haplotypes, with less common variants appearing generally only on less common haplotypes in a haplotype-specific manner. For example, KIR2DS1*005 (frequency = 0.004) is found only in haplotypes tB02 and tB07 while KIR2DS1*002 (frequency = 0.209) is found on tB01, tB03, and tB04 (Figure 3c). In contrast, many of the less frequent alleles of the inhibitory loci (e.g., KIR2DL4*002, KIR3DL1*007, KIR3DL1*008, KIR3DL2*011 and KIR3D3*003) appear on multiple haplotypes, including both the common and less common (Table S3). This is particularly evident at the framework loci, which exhibit the highest degree of overall variability.

### KIR Haplotype Frequencies

Table 1 details the estimated frequencies of the centromeric region, telomeric region, and full-length gene-content haplotypes. Haplotype frequencies in this cohort are very similar to those observed for a European white population [8]. Compared to those frequencies, our estimate for the Group A full-length haplotype is lower (54.1% vs. 62.5%), although the percentage of A haplotype centromeric or telomeric blocks is similar but distributed among haplotypes combining A and B structures (i.e., cA0x∼tB0x and cB0x∼tA0x). Haplotype frequencies within the centromeric or telomeric regions are also similar to those reported by Hou et al. [12], whose data set is a subset of that used here.

**Table 1:** Gene-content haplotype frequencies.

Centromeric/Telomeric Haplotype	Frequency	Full Haplotype	Frequency	D’
cA01	66.5%	cA01∼tA01	54.1%	0.22
cB02	17.1%	cB02∼tA01	14.3%	0.33
cB01	14.0%	cA01∼tB01	9.5%	−0.26
cB04	1.5%	cB01∼tA01	7.2%	−0.32
cB05	0.5%	cB01∼tB01	6.8%	0.36
cA02	0.3%	cB02∼tB01	2.8%	−0.16
cB03	0.1%	cA01∼tB05	1.8%	1
tA01	75.9%	cB04∼tB03	1.5%	1
tB01	19.4%	cA01∼tB06	0.8%	1
tB05	1.8%	cA01∼tB04	0.4%	1
tB03	1.5%	cB05∼tB01	0.3%	0.50
tB06	0.8%	cA02∼tB07	0.2%	1
tB04	0.4%	cB05∼tA01	0.2%	−0.47
tB07	0.2%	cA02∼tB02	0.1%	1
tB02	0.1%	cB03∼tA01	0.1%	1

Estimated frequencies for each centromere, telomere, and full reference haplotype. LD (D’) is included for each pairing of centromere and telomere. Haplotypes with frequencies less than 1% where determined by physical linkage and gene copy number of *KIR2DS4*.

Haplotype frequencies with allelic resolution are given in Table S3. The most common centromeric and telomeric allele-level haplotypes observed in this cohort are cA01- KIR3DL3*001∼KIR2DL3*002∼KIR2DL1C*002 (14%) and tA01- KIR2DL4*008∼KIR3DL1S1*3DL1*001∼KIR2DS4*003∼KIR3DL2*001 (14.0%), respectively.

Within the centromere, the cA01 structure dominates, with fifteen of the twenty most common haplotypes; the remaining five haplotypes are cB01 (n = 2) and cB02 (n = 3). Likewise, tA01 dominates in the telomere, with sixteen of the twenty most common haplotypes; the remaining four are tB01. When full-length (centromeric and telomeric) haplotypes are considered, thirteen of the top twenty are cA01∼tA01, three are cB02∼tA01, and there are two each of cA01∼tB01 and cB01∼tB01. These results are consistent with the findings of Hou et al. 2011 [12]. cA01∼tA01 is the only haplotype with a frequency (54.1%) greater than 15%; seven haplotypes have frequencies less than 1%.

LD statistics (D’) between the centromeric and telomeric regions are given in Table 1 (details are in Table S5). Complete LD between the centromeric and telomeric regions is observed in seven haplotypes, each of which has a frequency below 2%: cA01∼tB04, cA01∼tB05, cA01∼tB06, cA02∼tB02, cA02∼tB07, cB03∼ tA01 and cB04∼tB03. The remaining eight full-length haplotypes have much lower D’ values, between negative and positive 0.5; these include the six most common haplotypes in this cohort, observed at frequencies ranging from 2.8–54.1%.

**Figure 4:**
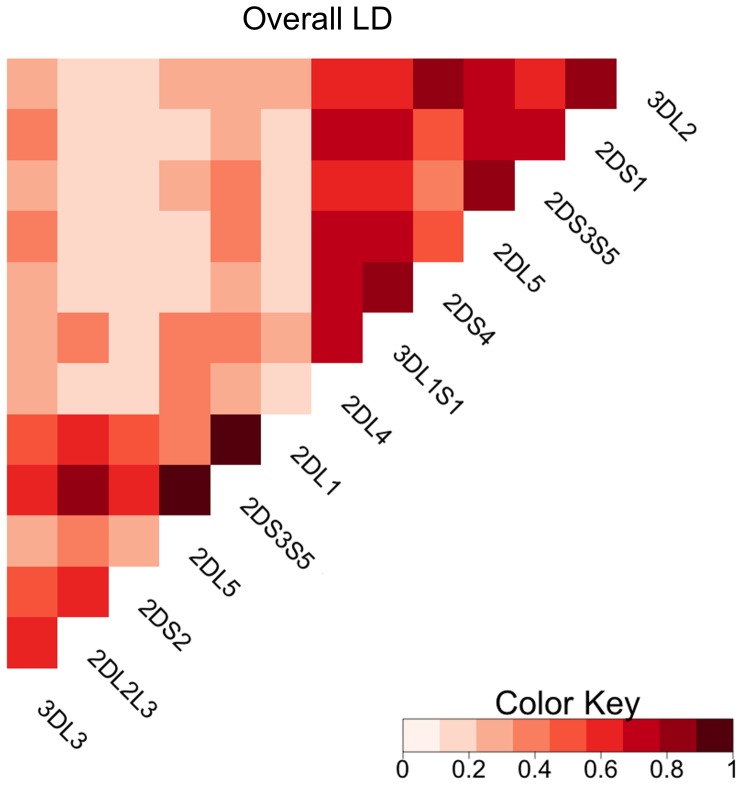
Overall linkage disequilibrium. Wn is displayed for each gene pair in a full-length KIR haplotype. Lighter shades indicate lower linkage disequilibrium and represent Wn approaching 0. Darker shades indicate higher linkage disequilibrium and represent Wn values approaching 1. Since they are now considered to be a single locus, *KIR2DL2* and *KIR2DL3* were combined into *KIR2DL2L3*; similarly, *KIR2DS3* and *KIR2DS5* were combined into *KIR2DS3S5.*

Overall LD between loci across the entire KIR region is depicted in Figure 4. As mentioned above, alleles of *KIR2DL2* and *KIR2DL3* are considered to be from a single locus, and thus were combined into *KIR2DL2L3* for simplicity of depiction. Similarly, *KIR2DS3* and *KIR2DS5* were combined as *KIR2D3S5*. Strong LD is observed within the centromere or telomere regions and minimal LD between the centromere and telomere regions; overall linkage disequilibrium between the genes flanking the intervening region is much lower (e.g., Wn = 0.17 for *KIR2DL1*∼*KIR2DL4*) than between the genes flanking each region (e.g., Wn = 0.46 for *KIR3DL3*∼*KIR2DL1* and 0.58 for *KIR2DL4*∼*KIR3DL2*). The highest overall LD is found between *KIR2DL5*∼*KIR2DS3S5* (in both the centromeric and telomeric regions), *KIR2DS3S5*∼*KIR2DL1* in the centromeric region, and *KIR3DL1*∼*KIR2DS4* in the telomeric region.

**Figure 5:**
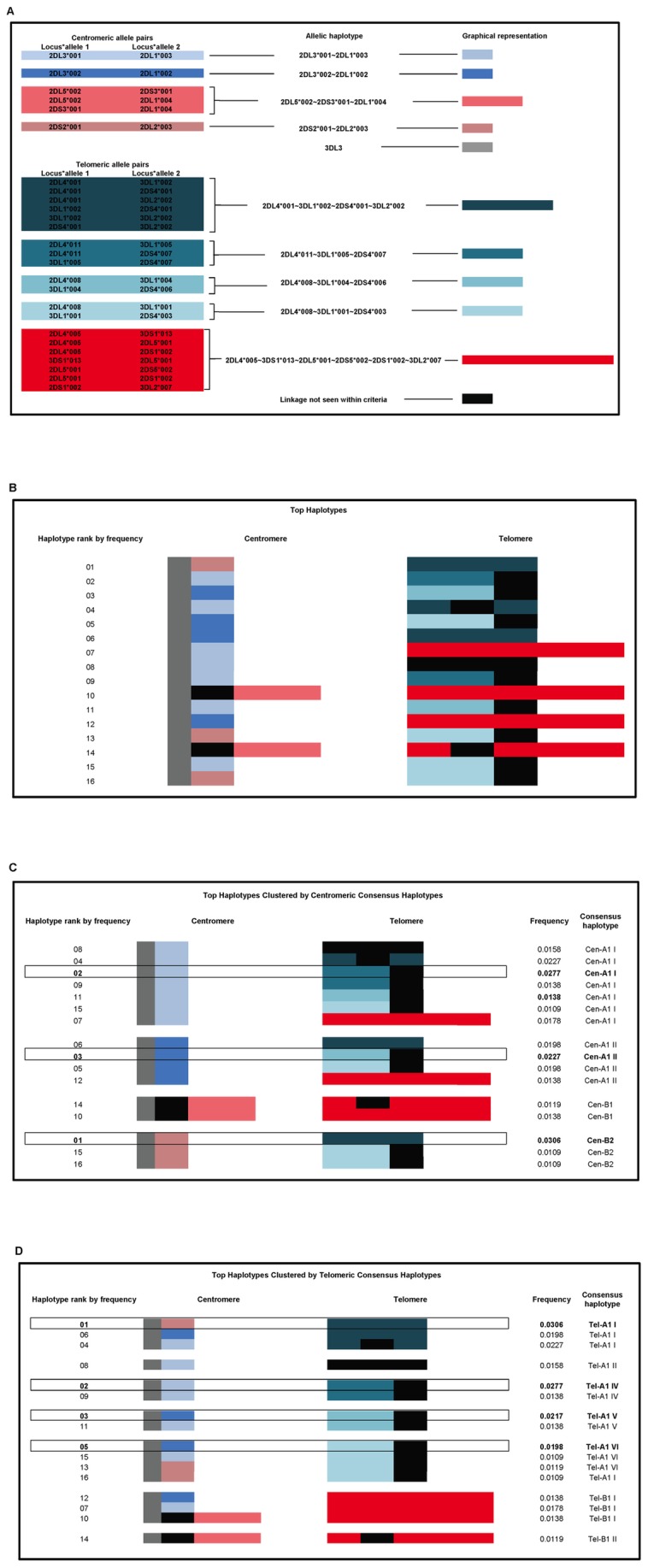
KIR gene haploblocks with fully resolved alleles at each locus. Allele pairs with high significance (p< = 0.0001), LD (D’ > = 0.9), and frequency (freq > = 0.1) are shown in column 1 (5a). Pairs found on the same haplotype are combined in column 2 and are represented by larger colored blocks in column 3. Pairs that don’t meet at least one of the three criteria are represented by black boxes. Full-length *KIR3DL3* genes are highly polymorphic and all alleles are represented as a single grey block. These blocks were mapped onto the 16 most frequent (freq >1%) allelic haplotypes and visually clustered by centromere (5b) and telomere (5c). The frequency within our sample and the name of the consensus haplotype (as defined by Hou et al. 2011) is shown for each haplotype. The haplotypes that are boxed and bolded emphasize that the most frequent haplotypes are generally distributed amongst different centromeric and telomeric clusters.

In order to examine the association of short haplotype blocks within the most common full-length haplotypes, the entire distribution of two-allele haplotypes was filtered on LD statistics (D’ > = 0.9; p< = 0.0001) and frequency (f > = 0.1), yielding a total of 26 common two-allele haplotypes that are observed in strong and significant LD (Figure 5a). These two-allele haplotypes were aligned with neighboring two-allele haplotypes to form longer allelic haplotype blocks. Contiguous two-locus haplotypes meeting these criteria are similarly colored in Figure 5a; two-allele haplotypes that do not meet the filtering criteria are represented in black. Figure 5b depicts the 16 most common (each with a frequency greater than 1%) full-length allelic haplotypes with allele pairs color-coded as in Figure 5a; these 16 haplotypes have a combined frequency of 28%. The haplotypes are numbered according to frequency, starting with ‘01′ for the most common haplotype. Figure 5c arranges the haplotypes based on centromeric allele content while in Figure 5d haplotypes are arranged based on telomeric allele content, illustrating the relative independence and likely frequent recombination between the centromeric and telomeric intervals of the KIR region. The haplotype blocks use the nomenclature and are consistent with those identified by Hou et al. [12]. In the centromere, the three most frequent full-length allelic haplotypes occur in different consensus haplotypes (identical centromeric allele blocks excluding *KIR3DL3*) and have a collective frequency of 8%. In the telomere, the four of the five most frequent full-length allelic haplotypes occur in different consensus haplotypes (identical telomeric allele blocks) and contribute 10% of all haplotypes. These haplotypes are boxed and bolded in Figures 5c and 5d.

The 506 individuals have 489 unique pairs of full-length allelic haplotypes; 17 haplotype pairs were seen twice (i.e., they occurred in exactly two individuals) and the rest are unique. Of the 17 pairs that occurred twice, 6 were structurally cA01∼tA01+cA01∼tA01, 4 were cA01∼tA01+cB02∼tA01, and the remaining 7 were unique.

**Figure 6:**
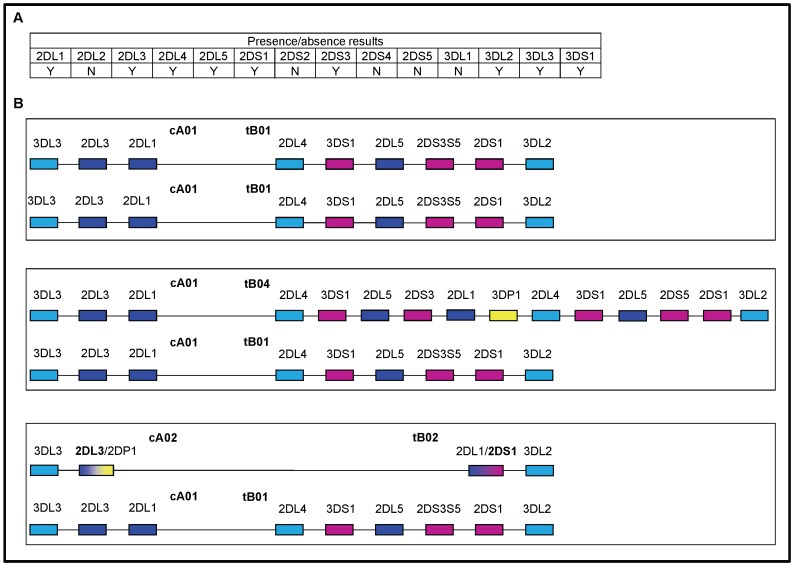
Structural genotypic ambiguity. Panel a depicts the presence or absence of specific loci in a particular genotype. Panel b illustrates three of the seven haplotype pairs that are consistent with the presence/absence genotype in panel a. The haplotype pairs contain 2, 3, and 1 copies of the *KIR3DS1*, *KIR 2DL5*, and *KIR2DS3S5* regions respectively.

205 of the 506 individuals have genotypes that are haplotypically ambiguous. Details are in Table S6 and Figure 6 provides a graphical example of such ambiguity.

## Discussion

This detailed study of KIR diversity, with the discovery of new alleles and haplotypes and the ascertainment of haplotype and allele frequencies using a multiplexed bioinformatics approach in a large European-American cohort, extends our understanding of the strength and extent of LD across the KIR region. To our knowledge, this is the first study of its kind to utilize multiple genotyping methods employed by different laboratories in order to verify allele calls for each individual in a cohort of this size. To ensure accurate results, each individual was genotyped to at least the three-digit allele resolution in multiple laboratories. 94.5% of the final genotypes were unambiguous at the three-digit level. Our careful laboratory analysis and consistent confirmation between laboratories further allowed identification of forty-seven novel alleles at nine loci in 63 individuals. While the sample cohort derives from successful matching for HLA in the context of hematopoietic stem cell transplantation and is therefore likely enriched for common HLA haplotypes, the large sample size and ethnic homogeneity, coupled with our redundant genotyping protocol, permit an unprecedented view of allelic variation across the KIR region. Assessment of the conformation of allele frequencies to expectations under Hardy-Weinberg equilibrium was performed for *KIR2DL2L3*– a locus expected to be carried by all haplotypes and one with the least ambiguous allele assignments. Due to ambiguities, alleles containing KIR2DL2*001 or KIR2DL2*005 were collapsed to a common allele; under this scenario, the proportions did not deviate from expectations under Hardy Weinberg Equilibrium (data not shown).

There are three sources of ambiguity in our data: structural, allelic, and phase (the knowledge of which genes occur together on a chromosome). The active recombination in the KIR region creates multiple large segments of unusual similarity, and thereby creates structural ambiguity by complicating gene identity and determination of physical location. Regions must often be physically linked with other regions of known location. The refinement over time of gene names in KIR nomenclature parallels this ambiguity. Allelic ambiguity is the result of typing with primers and/or oligonucleotides whose range and specificity don’t match all the regions or base pair combinations in a gene. Finally, current KIR typing methods don’t separate chromosomes, and therefore don’t specify phase.

In our study cohort, each of five full-length reference gene-content haplotypes occurs with a frequency over 6.8% and they collectively contribute 92% of the estimated haplotypes. The full-length reference haplotype frequencies largely conform to the frequencies reported by Pyo et al. [8], where six full-length reference haplotypes resolve 99% of genotypes in their sample of 96 European-American individuals. It is more difficult to compare our results with earlier studies [7], [11], [16], [56], [60], [61], [62], [63], [64], [65], [66], whose data were from dissimilar populations or were derived from lower resolution KIR genotyping results. However, we believe our results are consistent with the emerging understanding of content and diversity in the KIR region in its broadest context.

KIR haplotype estimation is complicated by gene-content and allelic variability and the inability of current typing methods to define this variability unambiguously. A primary structural complication is the occurrence of varying copy number of the same gene on a haplotype due to duplications or deletions. Haplotypes can be ambiguous when this copy number variation is not considered. Although most of the haplotypes are structurally unambiguous, ambiguity increases when haplotype pairs are considered. A primary factor is the apparent history of frequent recombination between centromeric and telomeric regions. For example, cA01∼tA01+cB01∼tB01 individuals (with an expected frequency of 7.4%) are identical for gene-content with cA1∼tB01+cB01∼tA01 individuals (1.4%). Gene copy number and insertions/deletions of varying length contribute more ambiguity: cA01∼tB01+cB02∼tA01 (predicted frequency of 2.7%) is indistinguishable from cA01∼tB05+cB02∼tB01 (0.10%). Similarly, a cA01∼tB01 homozygote containing two copies of the 3DS1∼2DL5∼2DS3S5 region has a structural genotype identical to the haplotype pair cA01∼tB01+cA01∼tB04, which contains three copies of the same region or the haplotype pair cA01∼tB01+cA02∼tB02, which contains one copy of the region (Figure 6). In fact, there are seven haplotype pairs that may explain the genotype that produces the cA01∼tB01 homozygous haplotype. A full list of haplotype pair ambiguities seen within our cohort can be found in Table S6. Given the parsimonious approach to full haplotype assignment and the fact that 92.5% of the samples did not undergo explicit linking and copy number analysis, our set of reference haplotypes might under-represent the true haplotype diversity of our cohort.

Due to copy-number variation for the KIR loci, allele-level haplotype estimates permit better characterization of allele frequencies than from genotypes alone. Analysis of KIR allele frequencies in our cohort reveals that the telomere is more diverse in terms of both gene-content and allelic variation than the centromere, with the exception of *KIR3DL3*, which is located in the centromeric region and is substantially diverse. The B haplotypes generally exhibit more structural (gene-content) variation but less allelic variation than the A haplotypes, as has been shown previously [9], [67].

This study serves as an example of how laboratory and computational methods can partner to illuminate details of a somewhat intractable genetic region. Initially, laboratories determine the major structural characteristics of the region; this produces a collection of structural reference haplotypes. Then, algorithmic approaches can fit sample genotypes into these reference haplotypes and flag individuals with potentially unknown structures. These two steps continue until all samples can be explained from a structural perspective. Subsequently, computational methods can leverage information inherent at the sample or population levels to resolve structural, allelic, and phase ambiguities in a maximum likelihood fashion. We anticipate that as genotyping methods for the KIR continue to mature, the reliance on computational methods will fall away. We expect this progression to continue as haplotype resolution expands to samples from other populations.

## Supporting Information

Table S1 Novel alleles identified. For each novel allele, the new name, most similar allele, altered codons, GenBank accession number, and number of individuals with the new allele is shown.
(DOC)

Table S2 2DL5/2DS3S5/2DL1 decision matrix for paralogous alleles. The genotypes represented in the first three columns (2DL5, 2DS3S5, 2DL1) are mapped to their centromeric and telomeric locations. The matrix contains and parsimoniously extends published associations [8].
(XLSX)

Table S3 Allelic haplotype frequencies. Allelic haplotypes and their frequencies, along with the structural haplotype assignment of each.
(XLSX)

Table S4 Allele frequencies. Absence of a locus is denoted by NNNN.
(XLSX)

Table S5 Regional haplotype frequencies. Frequencies of the centromeric and telomeric haplotypes separately and combined into full-length haplotypes. Also, D and D’ statistics for the full-length haplotypes.
(XLSX)

Table S6 Genotypes with ambiguous haplotype pair predictions. Each locus-level genotype is mapped to up to eight distinct structural haplotypes pairs.
(XLS)
