# Supplementary material for: Allele-Level Haplotype Frequencies and Pairwise Linkage Disequilibrium for 14 KIR Loci in 506 European-American Individuals
Source: PLoS One. 2012 Nov 5;7(11):e47491. doi: 10.1371/journal.pone.0047491 (PMC3489906; doi:10.1371/journal.pone.0047491)
Supplement: Table S1 — Novel alleles identified. For each novel allele, the new name, most similar allele, altered codons, GenBank accession number, and number of individuals with the new allele is shown. (DOC) [file pone.0047491.s001.doc]

**Supplemental Table 1.** Novel alleles identified**.** For each novel allele, the new name, most similar allele, altered codons, GenBank accession number, and number of individuals with the new allele is shown.

| **ID** | **Locus** | **Novel**  **Allele** | **Most Similar**  **Allele** | **Codons (Amino Acid) Altered** | **GenBank Accession**  **No.** | **No. individuals** |
| --- | --- | --- | --- | --- | --- | --- |
| NMDP-99 | 2DL1 | 020 | 00302 | 67 AGT ( S ) => GGT ( G ) | GQ421728 | 3 |
| NMDP-32 | 2DL1 | 021 | 002 | 106 GAG ( E ) => CAG ( Q ) | GQ421730 | 1 |
| NMDP-88 | 2DL4 | 00902 | 00901 | 137 GAG ( E ) => GAA ( E) | GQ421733 | 1 |
| NMDP-16 | 2DL4 | 014 | 00501 | 16 GCT ( A ) => ACT ( T ) | GQ412729 | 1 |
| NMDP-62 | 2DL4 | 015 | 00102 | 27 CGG ( R ) => TGG ( W ) | GQ421732 | 1 |
| NMDP-43 | 2DL4 | 016 | 00102 | 45 GTC ( V ) => TTC ( F ) | GQ421731 | 1 |
| NMDP-04 | 2DL5 | A*014 | A*0010101 | 167 GGA ( G ) => CGA ( R ) | GU063852 | 1 |
| NMDP-07 | 2DL5 | A*015 | A*0010101 | 114 CGC ( R ) =>TGC ( C ), 166 CAC ( H ) => CAT ( H ) | GU063853 | 1 |
| NMDP-112 | 2DL5 | B*016 | B*00201 | 78 CAC ( H ) => TAC ( Y ) | GU063851 | 1 |
| NMDP-73 | 2DS2 | 007 | 00101 | 10 CTC ( L ) => TTC ( F ) | GU063859 | 1 |
| NMDP-92 | 2DS2 | 008 | 00101 | 131 CGG ( R ) => TGG ( W ) | GU063860 | 1 |
| NMDP-68 | 2DS3 | 005 | 00103 | 47 GAC ( D ) => CAC ( H ) | GU063858 | 1 |
| NMDP-93 | 2DS5 | 00202 | 00201 | 158 AGA ( R ) => CGA ( R ) | GU063861 | 1 |
| NMDP-12 | 3DL1 | 00403 | 00402 | 218 AGC ( S ) => AGT ( S ) | GU063854 | 1 |
| NMDP-22 | 3DL1 | 072 | 00401 | 147 GTT ( V ) => ATT ( I ) | GU063855 | 1 |
| NMDP-55 | 3DL1 | 073 | 053 | 182 TCC ( S ) => ACC ( T ) | GU063857 | 1 |
| NMDP-46 | 3DL2 | 00104 | 00101 | 86 TCG ( S ) => TCA ( S ) | GU065736 | 1 |
| NMDP-57 | 3DL2 | 00702 | 00701 | 51 CAC ( H ) => CAT ( H ) | GU065739 | 1 |
| NMDP-79 | 3DL2 | 01102 | 01101 | 122 CAA ( Q ) => CAG ( Q ) | GU065740 | 1 |
| NMDP-104 | 3DL2 | 01103 | 01101 | 51 CAC ( H ) => CAT ( H ) | GU065732 | 2 |
| NMDP-38 | 3DL2 | 043 | 006 | 145 CGC ( R ) =>CAC ( H ) | GU065735 | 1 |
| NMDP-07 | 3DL2 | 032 | 00103 | 226 TGG ( W ) => CGG ( R ), 231 ATC ( I ) => ATG ( M ) | GU065733 | 1 |
| NMDP-56 | 3DL2 | 034 | 00701 | 237 GAA ( E ) => GGA ( G ) | GU065738 | 2 |
| NMDP-29 | 3DL2 | 042 | 00901 | 416 AGA ( R ) => TGA ( STOP ) | GU065734 | 1 |
| NMDP-51 | 3DL2 | 044 | 00103 | 190 GAC ( D ) => AAC ( N ) | GU065737 | 1 |
| NMDP-82 | 3DL2 | 045 | 00103 | 277 CGT ( R ) => TGT ( C ) | GU065741 | 1 |
| NMDP-85 | 3DL2 | 046 | 005 | 13 CGG ( R ) => TGG ( W ) | GU065742 | 1 |
| NMDP-97 | 3DL2 | 047 | 00101 | 73 AGA ( R ) => ACA ( T ),78 CGC ( R ) => CAC ( H ) | GU065743 | 1 |
| NMDP-100 | 3DL2 | 048 | 005 | 169 GCA ( A ) => TCA ( S ) | GU065744 | 2 |
| NMDP-02 | 3DL3 | 00104 | 00101 | 281 CAC ( H ) => CAT ( H ) | GU070841 | 2 |
| NMDP-30 | 3DL3 | 00209 | 00202 | 302 CAC ( H ) => CAT ( H ) | GU070845 | 2 |
| NMDP-83 | 3DL3 | 00210 | 00207 | 35 AAC ( N ) => AAT ( N ) | GU070853 | 1 |
| NMDP-78 | 3DL3 | 00302 | 00301 | 281 CAC ( H ) => CAT ( H ) | GU070851 | 1 |
| NMDP-108 | 3DL3 | 00904 | 00901 | 151 CAC (H ) =>CAT (H) | GU070856 | 1 |
| NMDP-81 | 3DL3 | 01002 | 01001 | 302 CAC ( H ) => CAT ( H ) | GU070852 | 1 |
| NMDP-49 | 3DL3 | 01308 | 01305 | 35 AAT ( N ) => AAC ( N ) | GU070847 | 2 |
| NMDP-111 | 3DL3 | 01309 | 01301 | 35 AAT ( N )=>AAC ( N ),62 GGC ( G )=>GGT ( G ) ,115 TCA ( S )=>TCG ( S ),238 GCG ( A )=>GCA ( A ),247 GCA ( A )=>GCG ( A ) | GU070840 | 2 |
| NMDP-61 | 3DL3 | 01407 | 01404 | 35 AAT ( N ) => AAC ( N ) | GU070849 | 1 |
| NMDP-25 | 3DL3 | 02703 | 02701 | 35 AAT ( N )=>AAC ( N ),115 TCA ( S )=>TCG ( S ),238 GCG ( A )=>GCA ( A ),247 GCA ( A )=>GCG ( A ), 281 CAT ( H )=>CAC ( H ) | GU070844 | 1 |
| NMDP-71 | 3DL3 | 036 | 020 | 128 AGG ( R ) => AGT ( S ) | GU070850 | 1 |
| NMDP-08 | 3DL3 | 041 | 00901 | 327 GCC ( A ) => CCC ( P ), 352 GAA ( E ) => GAT ( D ) | GU070842 | 6 |
| NMDP-20 | 3DL3 | 042 | 005 | 300 CAC ( H ) => AAC ( N ) | GU070843 | 1 |
| NMDP-40 | 3DL3 | 043 | 00902 | 79 CCA ( P ) => GCA ( A ) | GU070846 | 1 |
| NMDP-50 | 3DL3 | 044 | 00901 | 198 GGT ( G ) => GAT ( D ) | GU070848 | 2 |
| NMDP-95 | 3DL3 | 045 | 00301 | 287 AGT ( S ) => CGT ( R ) | GU070854 | 2 |
| NMDP-102 | 3DL3 | 046 | 00101 | 145 CGC ( R ) => CAC ( H ) | GU070855 | 1 |
| NMDP-110 | 3DL3 | 047 | 00301 | 17 GTG ( V ) => ATG ( M ) | GU070857 | 1 |
